# Program evaluation of a recuperative care pilot project

**DOI:** 10.1111/phn.12834

**Published:** 2020-11-15

**Authors:** Lauren Valk Lawson, Bonnie Bowie, Melanie Neufeld

**Affiliations:** ^1^ Seattle University College of Nursing Seattle WA USA; ^2^ Lake City Partners to End Homelessness Seattle Mennonite Church Seattle WA USA

**Keywords:** homelessness, program evaluation, public health nursing, recuperative care, respite care

## Abstract

**Objective:**

A program evaluation to demonstrate the feasibility of a recuperative care pilot project to address the needs of unhoused individuals.

**Design:**

The study is a descriptive postprogram evaluation.

**Sample:**

A total of 73 referrals were made to the project with 23 admissions.

**Measure:**

Data regarding number and type of referrals for admission, cost of respite care per guest and per day, hospital costs avoided, referrals to community services, and discharge destination were collected.

**Intervention:**

A case management care model was used. The project staff included a public health nurse and an outreach worker.

**Results:**

One local hospital accounted for 65% of all admissions. Admitting diagnoses were abscess/wound care (44%) followed by postsurgery recovery (17%). Housing resources (65%) was a common referral with 22% of guests discharged to stable housing. Actual length of stay exceeded the planned length by an average of 24 days. Total cost per guest per day was $157.45 which is an estimated savings to referring acute care facilities of between $18,000 and $48,000 per day.

**Conclusions:**

The project demonstrated an ability to provide unhoused individuals a place to recuperate following hospitalization in a cost‐effective manner. Challenges and recommendations of the program going forward were identified.

## 1 INTRODUCTION

The relationship between health and housing is well documented. Individuals who lack stable housing are at greater risk for morbidity and mortality when compared to housed individuals of the same age or gender. Common preventable ailments, such as hypertension, are exacerbated and can lead to chronic health problems when an individual lacks a safe place to rest, adequate nutrition, or a place to store medications (Fazel et al., 2014; Hwang et al., 2011; O'Connell, 2005). When an unhoused individual is hospitalized or has an illness requiring a period of recuperation, the lack of a place to recover hinders the healing process and the ability to convalesce. A growing response to the health needs of the unhoused is the development of medical respite care. Medical respite, as defined by the National Health Care for the Homeless Council (2016a), is the “acute and post‐acute medical care for homeless persons who are too ill or frail to recover from a physical illness or injury on the streets but are not ill enough to be in a hospital” (p.3). A significant benefit of medical respites is the reduction in length of hospital stays and decreased readmissions, thus, decreasing overall health care costs for this population (Buchanan et al., 2006; Doran et al., 2013).

A 2016 survey of existing medical respites in the U.S. revealed the majority provided short‐term housing with varying degrees of supportive services, such as case management. Case management addresses the whole person and focuses on access and utilization of services, such as substance abuse programs and assistance with permanent housing (NHCHC, 2016b). Additional services include care coordination with other service providers and preparations for discharge. The key to case management is an emphasis on developing the individual's abilities to meet their own self‐care needs, skills that are essential following discharge from the medical respite (Minnesota Department of Health, 2019). It was the decision to use a case management model that led to calling the program a recuperative care. According to the Health Resources and Services Administration, recuperative care services are similar to medical respites with a focus on case management instead of provision of medical services.

The recuperative care opened in May 2014 using a motel model which provided people, referred to as “guests”, with a safe space to heal while minimizing initial overhead costs. Along with the motel room, guest services included nursing care, case management, and other wraparound services, such as availability of nutritional foods, transportation, and advocacy to connect with other needed services. Staff included a public health nurse and an outreach worker. Trained volunteers provided check‐in support on weekends. Another volunteer was a local physician and member of the faith‐based organization. During the pilot project, the physician served on the advisory board and provided medical consultation. The overarching purpose of this project was to determine the feasibility of a motel respite care model for unhoused individuals upon hospital discharge. In addition, challenges to the program and recommendations for going forward were identified.

## 2 METHOD

This review is a descriptive evaluation to summarize the outcomes of the pilot project and discuss challenges and recommendations for future programs. All information was gleaned using content analysis of client files and the program financial spreadsheets kept during the pilot project. A documentation protocol instituted early in the development of the recuperative care assured standardization of files.

Data were collected on each guest using a detailed clinical intake form that included information such as the medical reason for hospitalization, mobility, mental health and/or substance abuse issues, ongoing treatment needs, and estimated length of stay in recuperative care (see Appendix A). Additional data were maintained in the standardized files by various staff throughout a guest's stay.

## 3 RESULTS

### 3.1 Referrals

Seventy‐three individuals were referred to the program during the 15‐month pilot project. Eight individuals, or 11% of referrals, were referred twice. Most of the referrals (86%) came from hospitals or clinics. Nine referrals (12%) came from community advocates, such as staff at a winter night shelter. One hospital was 2.5 miles from the recuperative care site and accounted for most of the referrals (63%) (See Table 1).

**TABLE 1:** Total referrals

Referent (miles from site)	Number of Referrals
Hospital A (2.5 miles)	46
Community Advocates	9
Hospital B (6 miles)	5
Hospital C (20 miles)	4
Hospital D (11 miles)	2
Single referrals from other sources	7
Total	73

### 3.2 Admissions

Once a referral was made, the public health nurse would triage the potential guest to assure a good fit with the recuperative care. Individuals needed to be able to provide their own self‐care and live independently in a motel room. After an introduction to the requirements of the program, a total of 23 people, or approximately one third of referrals, were admitted to the recuperative care program. Two individuals were admitted twice. The most common admitting diagnoses were 10 (44%) cases for abscess/wound care and 4 (17%) cases for postsurgery recovery. Referrals from Hospital A accounted for 65% of all admissions and represented 70% of the abscess/wound care admissions. Once a guest was admitted, a planned length of stay was determined based on the diagnosis, referral information, and an initial recuperative care assessment. The average length of stay per guest was 37 days with a range of 2 to 112 days.

Fifty referrals were declined over the 15‐month pilot period; the most common reason was lack of space (*N* = 12; 24%). Various other reasons for declining a referral were documented, the most significant was the acuity of medical needs (*N* = 7, 14%). As a recuperative care, guests needed to be able to perform their own activities of daily living in a motel room, which was an important consideration when admitting a guest. Another challenge was when referents were not available for intake, such as when the referent left the hospital against medical advice (*N* = 7; 14%). Five referrals were cancelled, two were declined because of the potential for disruptive behavior, and three did not respond to calls or did not show. An additional 14 individuals were not considered for admission, but limitations in early program documentation did not allow for analysis as to the reason.

### 3.3 Services received

Guests admitted to the recuperative care were provided with a motel room. Other services included case management, such as assistance with referrals to community resources. The most documented referral (65%) was to housing resources. Table 2 provides a breakdown of the types of referrals made for the guests. Additional case management services included assistance obtaining valid identification, such as state IDs, establishing primary care and legal services, and reconnecting to resources like Social Security and veteran benefits. Eleven guests required home health services when admitted to the recuperative care. These services were provided through a local home health agency. Of those referred to home health, a majority received wound care (91%), one received physical therapy, and another received diabetes education. Three guests were sent to an emergency department during their respite stay. Two were sent because of a decline in health. The third guest was seen in an emergency room because of a dog bite and was able to return to recuperative care.

**TABLE 2:** Documented referrals (*N* = 23)

Referral	Number of referrals
Housing resources	15
Primary Care Provider	7
Public Assistance	7
VA Services	3
Additional Rehab Services	3
Mental Health Services	3
Emergency Housing Screening	3
Total	41

### 3.4 Discharge destinations

Five or 22% of guests were discharged to stable housing situations. Four guests were discharged to a tent city, two to a hospital, one to a shelter, and one to jail. The remaining 30% were discharged without identified housing. See Table 3. The combined planned length of stay for all guests equaled 353 days, although the combined actual length was 901 days, a difference of 548 days. That amounts to an average of 24 days (3.4 weeks) beyond planned length of stay per guest. This total is reflected in the number of extensions granted to guests who needed additional recuperative services prior to discharge from the program. As noted earlier in this report, of the 23 guests, 17 (74%) were granted extensions within a range 1 to 11 extensions for a total of 55 weeks (mean = 2.5 weeks). The need for extensions demonstrated the complexity of the guests’ circumstances in terms of their health and social concerns. As noted above, most prominent was the number of guests needing wound care and assistance with finding stable housing.

**TABLE 3:** Discharge destinations (*N* = 23)

Discharge destination	Number of referrals
No Identified Housing	7
Tent City	4
Friend	3
ER/Hospital	2
Transitional Housing	2
Supportive Housing	2
Adult Family Home	1
Shelter	1
Jail	1
Total	23

### 3.5 Evaluation of pilot study costs

There were four patients with unusually long stays (85, 86, 109, and 114 days). If these outliers are omitted, the average daily per‐guest, per‐day direct care cost was $73.90 while the total per‐guest costs were $3,026.60. Costs per guess were averaged over the pilot project period. See Table 4 for an overview of guest costs. Administrative costs (overhead) were, on average, $5,790.84 per month during the 15‐month pilot and included personnel wages and benefits (Program Manager (administration) @ 0.5 FTE, Recuperative Care Coordinator (PHN) @ 0.5 FTE, Outreach Specialist @ 0.1 FTE (hired 4 months into the pilot project), bookkeeping @ 1 hr/month), office supplies and computer support, and training and travel expenses for staff. Daily administrative costs averaged $83.55 per patient. The total cost per guest per day was $157.45.

**TABLE 4:** Average cost of care

Expenses	Per Guest (*N* = 23)	Per Recuperative Care Day per Guest
Motel	$2,367.50	$57.81
Food	$318.31	$7.77
Transportation	$81.49	$1.90
Home Health	$224.38	$3.98
Medical Supplies	$51.59	$1.09
Other Supplies	$17.95	$0.34
Miscellaneous	$60.70	$1.01
Total Expenses	$3, 026.60	$73.90

### 3.6 Cost savings

During the pilot study, it was estimated that savings to the referring acute care facilities ranged between $18,000 and $48,000 per patient stay. Estimated costs were determined using predicted hospital costs for extended stays based on patient diagnosis. Table 5 compares the recuperative care costs with the estimated avoided hospital charges, or cost savings. Guest diagnoses upon admission are broken down into four diagnostic categories: wound care, medical diagnoses, surgical procedures, and infectious disease. Recuperative care costs include both direct care and administrative costs as described above for all guests in the diagnosis category. For example, in the wound care/cellulitis category, all costs for eight patients over their cumulative length of stays are included in the $50,047.47 total. The fourth column lists the estimated charges avoided, or cost savings, to the acute care facility by diagnostic category.

**TABLE 5:** Comparison of recuperative care costs and projected hospital charges avoided

Primary Diagnosis Category	Number of Guest	Total Recuperative Care Costs^a^	Estimated Number of Hospital Days per Guest	Estimated Total Acute Care Charges Avoided^b^
Wound Care/ Cellulitis	8	$50,047.47	7	$336,000
Surgical Procedure	4	$41,474.29	4	$96,000
Medical Diagnosis	7	$29,514.39	3	$126,000
Infectious Disease	4	$20,827.75	8	$192,000
Total	23	$141,863.80	125	$750,000
Per Guest Day	n/a	$157.45	n/a	$6,000

^a^ Includes administrative costs of $83.55 per patient day.

^b^ Based on referring hospital staff estimated length of stay and charges per day.

What is not included in Table 5 is the potential lost revenue to hospitals when patients are not discharged in a timely manner. At the time of the pilot study, most hospitals in the Puget Sound area were at or over capacity much of the time and could have benefitted from an alternative place to transfer patients extended beyond the typical length of stay due to lack of housing.

### 3.7 Challenges and lessons learned

Some challenges encountered while analyzing the information included inconsistent and evolving documentation practices. Often this was the result of changes in staff. Insight from the review process will help to improve the documentation when planning to re‐open the recuperative care program. As an example, an electronic form of documentation would help improve consistency.

Early lessons learned informed the changes to admission process. With experience it became easier for the public health nurse to triage which individuals would be most appropriate for independent living in a motel room. The opportunity to interview potential guests while still in the hospital provided better assessment of a person's fit for the program. These experiences also helped to clarify with the referring agencies who the respite care had the capacity to take.

Longer length of stays than initially planned proved a challenge. Navigating the housing systems and availability of affordable housing was often a factor. A future consideration is to have a staff member entirely devoted to housing assistance.

Another area for improvement is to develop a better system to track people once they are discharged. Closer follow‐up would provide better information regarding guest continuation with resources and access to housing, two important aspects of case management in recuperative care.

Finally, a lack of clarity regarding the type of housing guests went to upon discharge limited the capacity of the study to demonstrate an ability to transition guests into stable housing. As an example, the percent housed could be increased to 35% if guests discharged to stay with a friend qualified as a stable living situation. Unfortunately, being “doubled up” or temporarily living with a friend is not considered a stable housing situation and, in some circumstances, can still qualify a person as homeless. Clearer information on discharge destinations would provide better evidence of peoples’ ability to transition out of homelessness following recuperative care.

## Appendix A

### JustHealth Recuperative Care Pilot Referral Form

To refer a patient from your hospital, please complete the entire form and fax to:

**Referring Hospital/Clinic:** Floor/Unit:

**Attending Physician:** Phone #: Pgr:

**Primary Care Provider/Clinic:** Phone #:

**Discharge Planner:** Phone #: Pgr:

**Authorized by:** Date: (For Internal Hospital Use)

If Medicaid: Managed Care Program: __CHPW __Molina __Amerigroup __Coordinated Care

### Patient Information

**Name**

**Date of Birth: Anticipated Discharge Date**

Date Admitted to Hospital:

___Patient is agreeable to Recuperative Care Admission

___ If in ETOH withdrawal, CIWA < 10 for 16 hours without benzodiazepine meds.

___Independent in mobility, transfers and feeding, not a known fall risk

___Patient has a medical need requiring recuperative care

**Reason for requiring Recuperative Care**

**Medical reason for hospital admission (include ICD code)**

Acuity:

Surgical procedures and/or patient limitations:

Home Health? __Y __N If Yes, explain:

Wound Care? __Y __N If Yes: Size ____cm by ____cm Depth____cm Stage___

Oxygen? __Y__N Diabetic? __Y __N

Labs? __Y __N Coumadin? __Y __N

IV Antibiotic? __Y __N Communicable disease? __Y __N

PT/OT? __Y __N Insulin dependent? __Y __N

**Does the patient have any mental or substance abuse issues?**

**Mental Health:** __ Bipolar __ Depression __ Suicidality __ Schizophrenia __ Other:

**Substance Abuse:** __ Alcohol __ Cocaine __ Heroin __ Methamphetamine __ IV Drug use __ Other:

**Any other medical or behavioral problems?**

**Self‐administer medicine?** __Y __N If no: __Needs reminders __Needs assistance

**Continent (bowel & bladder)?** __Y __N Explain:

**Ambulatory?** __Y __N If no: __Cane __ Crutches__ Walker __ Wheelchair __ Other:

**Special Diet?** __Y __N Explain:

**Medications List (please fax)**

___ 30 day supply of medication

____ 1 week supply of dressings

____ 3 day supply of narcotics

**Estimated length of stay in recuperative care center: ____ days**

____ STAT discharge summary (must be faxed prior to patient's arrival)

____ Home Health arranged

___IV antibiotics with Infectious Disease Follow‐up

**Follow‐up**

FOR RECUP Staff Use Only

**Approved?** __Y __N If denied, reason: **Admission Date: Time:**

Reviewed by: Date: Time: **Discharge Date: Time:**

Language: Patient Summary Y__ N___
